# Geospatial modelling for zoonotic disease hotspot identification within a One Health framework: a systematic review

**DOI:** 10.1186/s42522-026-00194-8

**Published:** 2026-01-27

**Authors:** Jabulani Nyengere, Willard Mbewe, Lucius Malalu, Harineck Tholo, Allena Laura Njala, Takondwa Sembo, Sylvester William Kumpolota, Richard Lizwe Mvula, Chikondi Chisenga, Charity Kanyika-Mbewe, Alfred Maluwa, Fasil Ejigu Eregno

**Affiliations:** 1 Ndata School of Climate and Earth Sciences, Malawi University of Science and Technology, P.O Box 5196, Limbe, Malawi; 2 Department of Education Sciences, University of Livingstonia, P. O. Box 112, Mzuzu, Malawi; 3 Faculty of Engineering Science and Technology, UiT The Arctic University of Norway, Postboks 385, Narvik, 8514 Norway

**Keywords:** Geospatial modelling, Zoonotic disease, One Health, Hotspot analysis, Disease mapping

## Abstract

Zoonotic diseases continue to pose significant public health threats worldwide, driven by complex interactions at the human–animal–environment interface. Geospatial modelling has emerged as a critical tool for identifying disease hotspots and supporting One Health–oriented surveillance and intervention strategies. However, a systematic synthesis of how geospatial approaches operationalize One Health principles remains limited. A systematic review was conducted following PRISMA 2021 guidelines to synthesise peer reviewed studies published between 2000 and 2025 that applied geospatial modelling to identify zoonotic disease hotspots. Multiple bibliographic databases were searched, and studies were screened using predefined inclusion criteria. Data were extracted on modelling approaches, predictor variables, geographic focus, and levels of One Health integration, followed by qualitative and quantitative descriptive synthesis. A total of 46 studies met the inclusion criteria. Publication output increased markedly after 2020, with studies concentrated in Africa, Asia, and Europe. Bayesian spatial models, satellite imagery–based analyses, machine learning methods, and ecological niche modelling were most frequently employed. Climatic variables dominated predictor selection, while socio ecological and animal health variables were less consistently integrated. Full integration of human, animal, and environmental domains was observed in only 15.2% of studies, with most exhibiting partial or implicit alignment with One Health principles. Data availability, quality, and spatial and temporal resolution were the most reported limitations. Geospatial modelling plays an increasingly important role in zoonotic disease hotspot identification, yet its capacity to operationalise One Health remains constrained by data fragmentation and uneven domain integration. Strengthening integrated surveillance systems, expanding socio ecological predictor inclusion, and promoting harmonised methodological standards are essential for enhancing the policy relevance and operational impact of geospatial approaches in zoonotic disease prevention and control.

**Supplementary Information:**

The online version contains supplementary material available at 10.1186/s42522-026-00194-8.

## Introduction

The One Health approach has gained increasing prominence as an integrative framework for addressing complex health challenges at the interface of humans, animals, and the environment. Recognizing that diseases transmitted between humans and animals (zoonotic) emerge from interconnected ecological and socio-economic systems, One Health emphasizes interdisciplinary collaboration and evidence-based decision-making to safeguard population health across sectors [1, 2]. This integrative paradigm has become particularly essential in low- and middle-income countries where human–livestock–environment interactions are intensified by land-use change, climate variability, and rapid population growth. As global health systems confront the rising risks posed by emerging and re-emerging pathogens, a spatially explicit understanding of disease transmission dynamics is increasingly necessary for surveillance, prevention, and policy design [3]. Unlike existing reviews that broadly examine spatial epidemiology or zoonotic disease mapping, this review focuses specifically on geospatial hotspot modelling as a decision-oriented analytical approach within One Health systems. It emphasizes how spatial models are used to identify zones of risk convergence where human exposure, animal reservoirs, and environmental suitability intersect, rather than merely describing disease distributions. In addition, the review explicitly examines how One Health principles are operationalised through the integration of multi-domain data and spatio-temporal modelling frameworks. By synthesizing methodological advances, predictor integration, and practical applications across diseases and regions, this review provides a distinct contribution to understanding how geospatial modelling supports actionable, systems-based One Health surveillance and intervention planning.

Communities across Africa and other vulnerable regions continue to face recurrent disease infestations that stem from both endemic and emerging zoonoses. These include infections such as anthrax, brucellosis, Rift Valley Fever, and other vector-borne or environmentally mediated diseases that disproportionately affect rural populations [4, 5]). The spatial clustering of such diseases is strongly influenced by environmental conditions, livestock production systems, land-use patterns, wildlife movements, and socio-economic vulnerabilities [6]. Mapping the geographic distribution of these risks is therefore critical for implementing timely and targeted One Health interventions, strengthening surveillance systems, and enhancing early warning capacities. Spatial insights also support local authorities and public health practitioners in optimizing resource allocation and tailoring community-level prevention strategies. While the burden of zoonotic diseases and the urgency of One Health implementation are particularly pronounced in Africa and other low- and middle-income countries, this review adopts a globally inclusive scope. Studies from all geographic regions were considered to capture the full range of geospatial modelling approaches, data integration strategies, and operational contexts. The emphasis on high-burden and resource-constrained settings reflects their disproportionate vulnerability and the methodological challenges they present, rather than a geographic restriction of the review.

Given the spatial heterogeneity of zoonotic disease transmission, geospatial modelling has become a central tool for identifying and predicting disease hotspots. Advances in geographic information systems, remote sensing, ecological niche modelling, and machine learning have facilitated the development of sophisticated spatial models capable of capturing multi-layered drivers of disease risk [7, 8]. Recent studies have applied these models to map the risk distribution of diverse zoonoses, evaluate environmental suitability for vectors, and understand interactions within complex socio-ecological systems. Geospatial methods have therefore become indispensable not only for scientific inquiry but also for guiding public health responses, veterinary interventions, and environmental management within One Health frameworks.

**Fig. 1:**
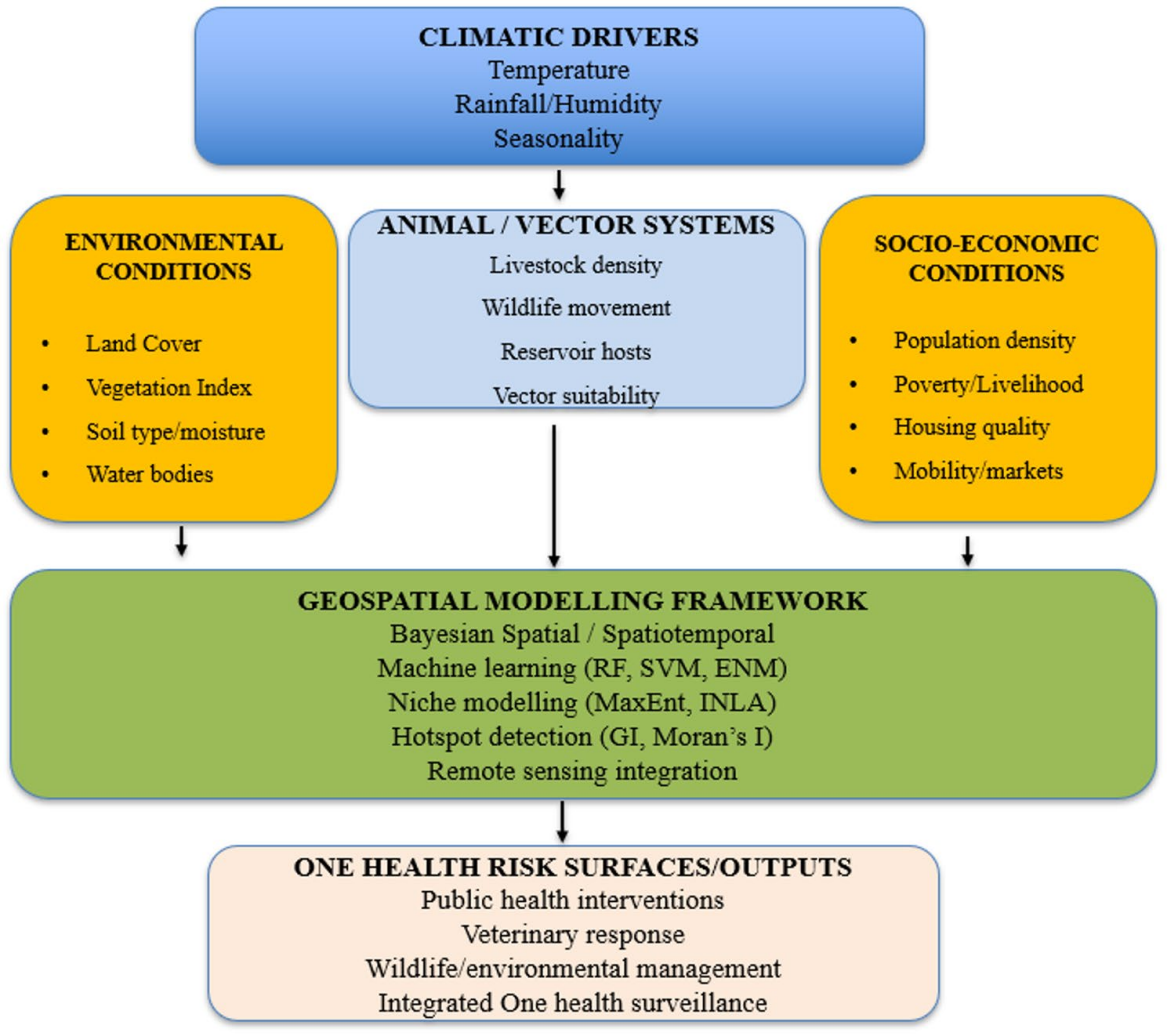
Conceptual diagram of One health geospatial framework

Although geospatial modelling has advanced rapidly and is now widely applied across human, animal, and environmental health research, the underlying pathways through which environmental, climatic, animal, and socio-economic drivers interact to shape zoonotic disease risk are often described in fragmented ways in the literature [3, 7]. Existing studies typically emphasize isolated components, such as ecological suitability, vector dynamics, or human exposure patterns, without presenting an integrated representation of how these domains converge within One Health systems to influence spatial patterns of disease emergence [6, 9]. To address this gap, and to clarify the theoretical foundation guiding this review, a conceptual framework is presented to illustrate the multi-domain interactions that geospatial models seek to capture (Fig. 1). This framework synthesizes the core determinants of zoonotic transmission and demonstrates how their integration supports the identification of hotspots, spatial clustering, and transmission corridors within a One Health context [1, 2].

The One health conceptual framework presented in Fig. 1 exemplifies the integration of environmental, climatic, animal, and socio-economic factors, framed within the One Health approach to identify zoonotic disease hotspots. It highlights how environmental variables, such as land cover, vegetation indices, soil type, and proximity to water, underpin pathogen and vector survival [7, 8]. Studies have documented that climatic factors, such as temperature, rainfall, and humidity, influence vector behavior and pathogen replication, thereby acting as critical predictors in spatial risk assessments [3, 10].

Animal-related determinants, like livestock densities and wildlife movements, play a role in interactions that sustain pathogens and promote spillover mechanisms [1, 6]. On the other hand, socio-economic elements, such as population density, human mobility, poverty, and housing conditions, are very key shaping exposure and vulnerability to zoonotic diseases ([4, 5]. These diverse datasets are integrated into geospatial modelling platforms utilizing Bayesian spatial models, machine learning techniques, ecological niche modelling, and hotspot detection algorithms to create probabilistic risk surfaces [9, 11]. This integration facilitates the generation of high-resolution hotspot maps, delineates transmission corridors, and provides early-warning signals that aid coordinated decision-making across human health, veterinary fields, and environmental management [2].

Although many studies have explored spatial modelling of zoonotic diseases, the evidence base remains fragmented across pathogens, ecosystems, and methodological approaches. Several investigations have focused on single-disease mapping, local-scale modelling, or specific environmental determinants, yet there is a lack of consolidated synthesis that integrates geospatial modelling evidence from a One Health perspective [9, 10]. The absence of a comprehensive review limits understanding of how spatial tools have been applied across disciplines, where methodological gaps persist, and how geospatial evidence can better inform integrated One Health decision-making. A systematic review is therefore warranted to examine how geospatial modelling has been utilized to identify zoonotic disease hotspots, evaluate methodological advances, and highlight opportunities for strengthening multi-sectoral surveillance systems.

The relevance of synthesizing this evidence lies in its potential to guide policymakers, health practitioners, and researchers toward effective utilisation of spatial tools within One Health programming. By consolidating existing geospatial studies, the review will help clarify dominant modelling approaches, identify data gaps and limitations, and highlight regions or pathogens that require enhanced surveillance. Such insights are crucial for improving risk assessment frameworks, designing integrated health strategies, and supporting early warning and intervention planning amid growing global health threats.

The objective of this systematic review is to examine the application of geospatial modelling techniques for identifying zoonotic disease hotspots within a One Health framework. Specifically, the review aims to assess the methodological approaches used in spatial modelling of zoonotic diseases, evaluate how One Health principles have been incorporated into geospatial analyses, and synthesise evidence on spatial determinants that drive disease clustering across human, animal, and environmental systems.

## Methodology

### Review protocol

This systematic review followed the Preferred Reporting Items for Systematic Reviews and Meta-Analyses (PRISMA) 2021 guidelines to ensure methodological transparency, reproducibility, and scientific rigor [12]. The protocol was developed prior to the literature search and defined the objectives, research questions, search strategies, inclusion and exclusion criteria, and quality appraisal procedures. Given the multidisciplinary nature of One Health, the review was designed to capture literature from human health, veterinary science, environmental science, and spatial epidemiology domains. The protocol guided the review through three phases: planning, conducting, and reporting, aligned with established systematic review standards in geospatial modelling and public health research [13, 14]. Although the review protocol was developed a priori to guide the search strategy, study selection, and synthesis, it was not formally registered in an international repository such as PROSPERO or OSF. Nevertheless, all methodological steps were defined in advance and adhered to in accordance with PRISMA 2021 recommendations.

### Planning stage

**Fig. 2:**
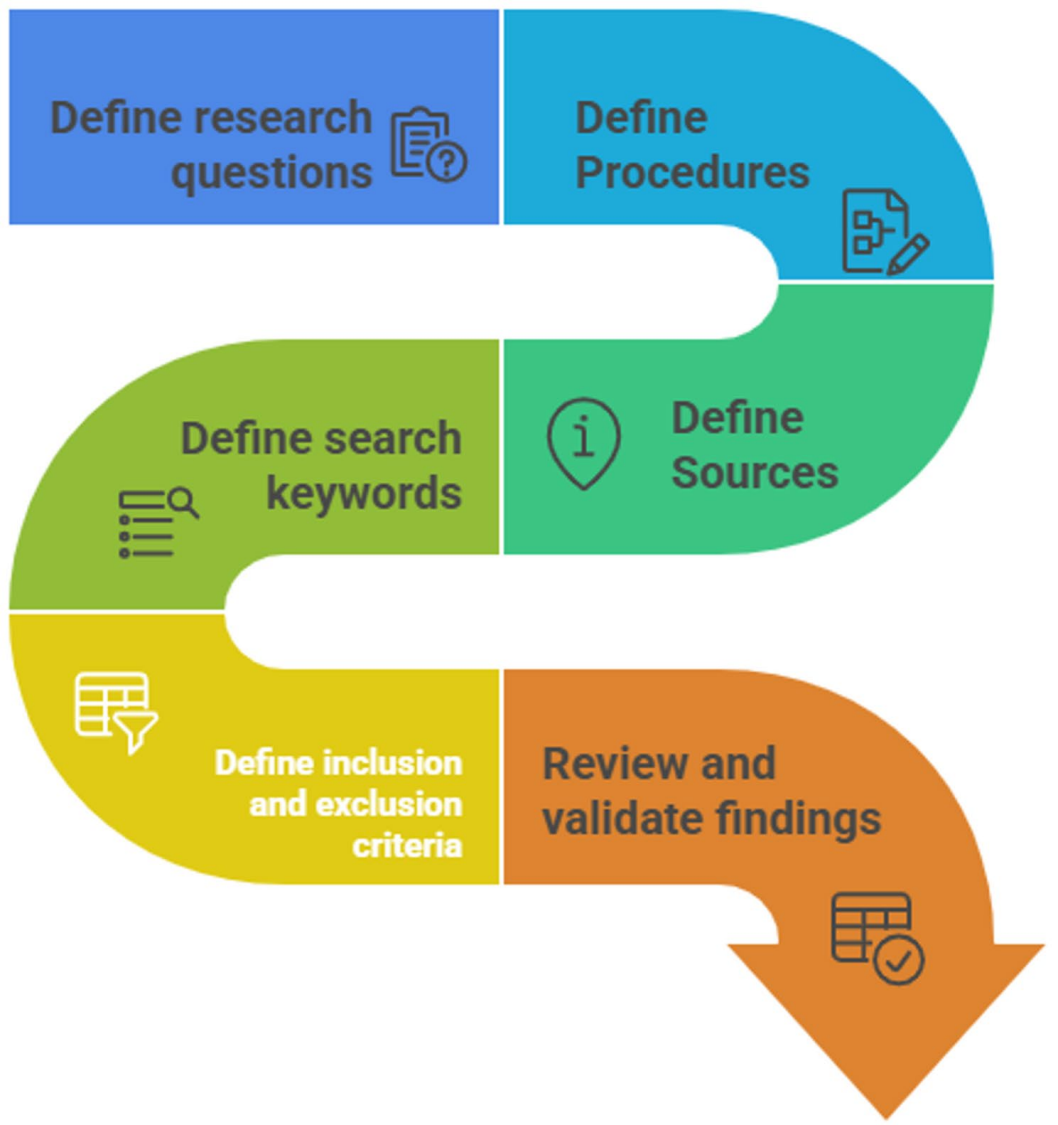
The plan review step by step process

During the planning stage (Fig. 2), the research team refined the central review question to explicitly investigate how geospatial modelling has been applied to identify and characterize zoonotic disease hotspots within a One Health framework. This guided the drafting of a comprehensive search strategy that incorporated specific terminology related to zoonotic diseases, geospatial techniques, and One Health concepts. In accordance with the protocol of the reference manuscript, this planning stage concluded with an iterative process of testing and refining the search terms across multiple databases to ensure a comprehensive and sensitive retrieval of relevant literature.

### Conducting stage

#### Literature search

Systematic searches were performed across eight major scientific databases selected for their relevance to public health, veterinary epidemiology, and environmental sciences: ScienceDirect, PubMed, MDPI, JMIR, Wiley online library, Research gate, Springer Link, and Google Scholar. To capture the full spectrum of geospatial zoonotic disease modelling, Boolean combinations of keywords were used. The final search string applied across databases was: (“zoonotic disease” OR “zoonosis” OR “zoonoses”) AND (“geospatial modeling” OR “GIS” OR “spatial analysis” OR “hotspot mapping” OR “spatial epidemiology” OR “remote sensing”) AND (“One Health”). Database-specific adjustments were made where required. Search results were exported into Mendeley and Microsoft Excel for de-duplication. Google Scholar and ResearchGate were included as supplementary search platforms to capture relevant peer-reviewed studies that may not be comprehensively indexed in conventional bibliographic databases, particularly within interdisciplinary One Health and geospatial research. To ensure reproducibility and consistency, searches on these platforms followed a structured and predefined procedure, with results screened based on relevance ranking and explicit stopping rules, as detailed in the Supplementary Material. Database-specific search strategies were adapted to each platform’s syntax and indexing requirements. Full search strings, including Boolean operators, field restrictions, and applied filters for all databases, are provided in the Supplementary Material to ensure reproducibility. For MDPI, an additional Africa-focused filter was applied as a supplementary search strategy to ensure adequate representation of studies from high-burden and historically underrepresented settings within One Health and geospatial research. This filter was not intended to restrict the overall geographic scope of the review, which remained globally inclusive, but rather to complement unrestricted database searches and mitigate potential indexing bias. This section describes the execution of the literature search across selected databases, following the predefined and iteratively refined search strategy outlined in Sect. 2.6.

#### Screening process

Study selection was executed in two distinct and rigorous stages, following the established three step screening model used in the reference one health systematic reviews. The first stage involved a dual independent review of all retrieved records by two reviewers, who performed a title and abstract screening against the predefined inclusion and exclusion criteria. This was followed by the second stage, a comprehensive full text evaluation of all potentially relevant articles. During this full text assessment, reviewers independently evaluated each study for its methodological clarity, its explicit or implicit relevance to the One Health framework, and the appropriateness of its applied geospatial modelling techniques. To ensure objectivity and consistency throughout this process, any disagreements between the two independent reviewers at either stage were systematically resolved through discussion until a consensus was reached.

#### Inclusion and exclusion criteria

The inclusion criteria required that selected publications: focus on a zoonotic disease or pathogen; employ a geospatial methodology (e.g., modelling, GIS, remote sensing, or spatial statistics); explicitly or implicitly align with One Health principles by integrating human, animal, and/or environmental dimensions; be published in English in a peer-reviewed journal between 2000 and 2025; and provide sufficient methodological detail for evaluation. Consequently, studies were excluded if they lacked a geospatial analytical component, were primarily descriptive without formal modelling, focused exclusively on non-zoonotic diseases, or did not meet the threshold for methodological transparency. Alignment with One Health principles was assessed using predefined operational criteria to reduce subjectivity. Studies were classified as *explicitly* aligned if they directly referenced One Health and integrated data or analysis from at least two of the three core domains (human, animal, and environmental health). Studies were classified as *implicitly* aligned if they did not explicitly reference One Health but incorporated geospatial modelling that analytically linked environmental drivers with human or animal disease outcomes in a manner consistent with One Health systems thinking. Studies that addressed only a single domain without cross-sectoral linkage were excluded. No geographic restrictions were applied during the literature search or study selection process; eligible studies from all global regions were included, allowing comparative insights across diverse epidemiological, environmental, and socio-economic contexts. Grey literature, including reports, theses, preprints, conference abstracts, and non–peer-reviewed materials, was excluded from the review.

#### Quality assessment

Quality appraisal employed a structured evaluation tool adapted from key spatial epidemiology guidelines (Franco et al., 2021; Rahman et al., 2023) to systematically assess each study’s methodology. The assessment criteria encompassed the clarity of the research objectives, the adequacy and description of the geospatial data sources, the thoroughness in detailing the chosen modelling techniques, and the extent of meaningful integration of One Health concepts. Furthermore, each study was evaluated for the validity of its specific spatial analysis including the application of hotspot algorithms, kernel density estimation, or suitability modelling, and for the transparency with which it presented its results and acknowledged its limitations. Any study that did not meet the established minimum methodological threshold across these critical domains was subsequently excluded from the final synthesis during this rigorous screening stage. The degree of One Health integration was further assessed during quality appraisal using the same predefined criteria, focusing on the extent of cross-domain data integration, analytical coupling within models, and interpretation of results in a multisectoral context. Quality appraisal was conducted using a structured scoring framework adapted from established spatial epidemiology guidelines. Each study was assessed across four core domains: (i) clarity of objectives and study design, (ii) adequacy and transparency of geospatial data sources, (iii) appropriateness and rigor of spatial modelling methods, and (iv) integration of One Health principles and interpretation of results. Each domain was scored on a three-point scale (0 = not addressed or unclear; 1 = partially addressed; 2 = adequately addressed), yielding a maximum possible score of 8. No differential weighting was applied across domains. Studies scoring below 4 were considered to fall below the minimum methodological threshold and were excluded from the final synthesis. Quality assessment informed inclusion or exclusion and guided interpretation of results, but no formal weighting of findings based on quality scores was applied during synthesis.

#### Data extraction and synthesis

A structured extraction template, consistent with the approach in the reference manuscript, was used to extract variables including author and year, country and sub region, the specific zoonotic disease or pathogen type, the geospatial modelling approach employed, the environmental, climatic, and socio ecological predictors used, the data sources, key results such as identified hotspots and risk factors, and the alignment with One Health principles. The subsequent synthesis was conducted both descriptively, through frequency distributions, and thematically, focusing on modelling strategies, predictor categories, and the reported strengths and weaknesses of the studies.

### Reporting stage

The reporting stage followed PRISMA 2021 standards. A PRISMA flow diagram was produced to illustrate the number of studies identified, screened, excluded, and retained for review. Reporting emphasized the extent and characteristics of geospatial modelling efforts highlighting methodological trends and gaps.

**Fig. 3:**
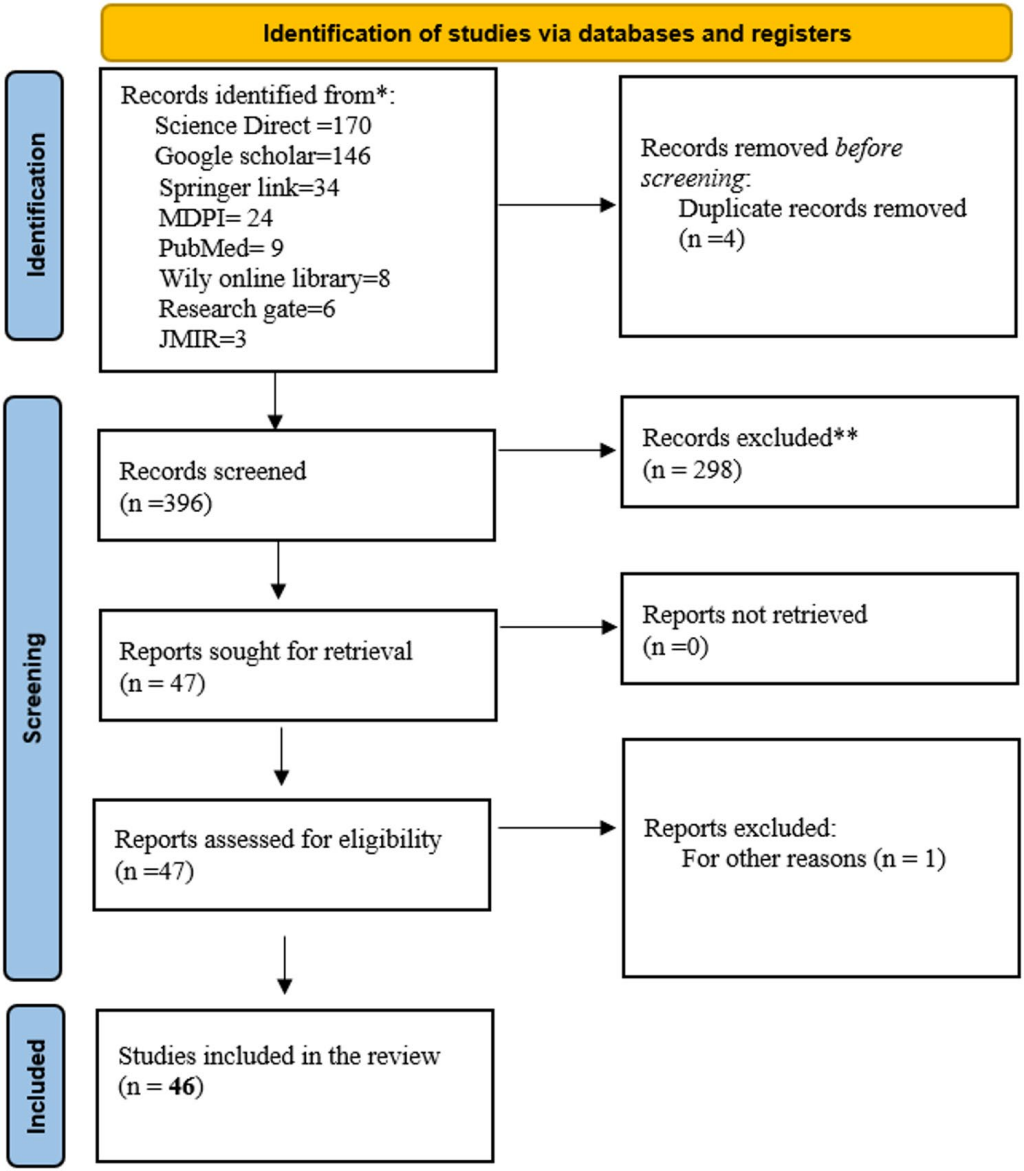
PRISMA flow diagram utilized in the study

### Research questions

This review addressed four research questions:

RQ1: Which geospatial modelling approaches have been employed to detect zoonotic disease hotspots?

RQ2: Which environmental, climatic, animal health, and socio-ecological variables are most commonly used as predictors?

RQ3: How have One Health principles been integrated into geospatial modelling frameworks?

RQ4: What methodological, data, and contextual challenges constrain geospatial modelling of zoonotic diseases across different geographic and epidemiological contexts?

### Search strategy

The search strategy was developed iteratively through exploratory searches and refinement of keywords to ensure sensitivity and specificity across disciplines. This section describes the process used to construct and optimize the search terms, while the execution of the final search is detailed in Sect. Literature Search. Synonyms for zoonotic pathogens, modelling techniques, and One Health concepts were incorporated to maximize coverage. Database-specific search strategies were applied, which included:

- ScienceDirect: “zoonotic disease” AND “GIS hotspot modelling”.
- PubMed: (“zoonoses”[MeSH]) AND “spatial epidemiology”.
- MDPI: full Boolean expression with filters for Africa.
- SpringerLink: “spatial analysis” AND “One Health”.
- Google Scholar: broad keyword search to capture grey peer-reviewed literature.

All searches were restricted to peer-reviewed studies.

## Results

### Distribution of papers

The initial database search yielded a total of 400 articles from eight major academic sources (Fig. 3). Following the rigorous application of the predefined inclusion criteria during the title, abstract, and full text screening stages, 46 articles were ultimately deemed eligible for final inclusion in the systematic review. The distribution of these eligible studies across sources was 16 from Science Direct, 13 from Google Scholar, 11 from Springer Link, 3 from MDPI, and one article each from JMIR, PubMed, and Wiley Online Library. No articles sourced from Research Gate met the necessary methodological and thematic criteria for inclusion.

#### Distribution of reviewed papers based on year

**Fig. 4:**
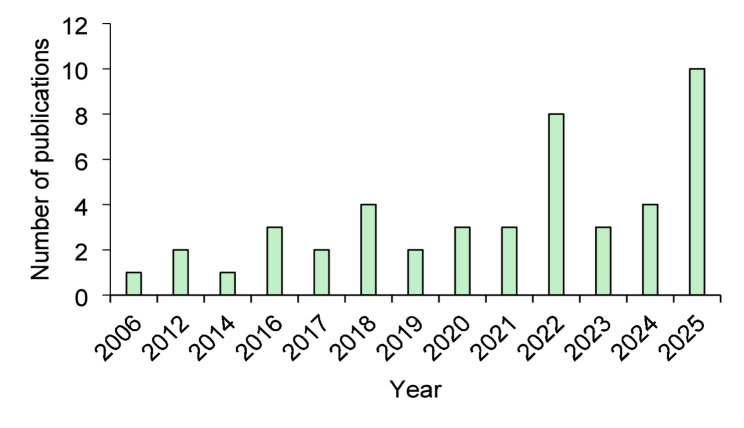
Distribution of the selected publications per year

The annual distribution of the selected publications, illustrated in Fig. 4, reveals a significant and accelerating trend in the application of geospatial modelling to zoonotic disease hotspots within a One Health context. Publication trends reveal a clear temporal progression. During the early period (2000–2014), publications were sparse, with fewer than two studies per year. A modest increase was observed in the mid-period (2015–2019), coinciding with growing use of geospatial tools in infectious disease research. Following the onset of COVID-19 (2020–2022), publication frequency increased markedly, reflecting heightened interest in spatial epidemiology and zoonotic risk. The most pronounced growth occurred in the recent period (2023–2025), during which nearly half of all included studies were published. This trend finished in a pronounced peak of ten publications in 2025, as depicted in Fig. 4, indicating a marked recent increase in publications.

#### Distribution of reviewed papers by country

**Fig. 5:**
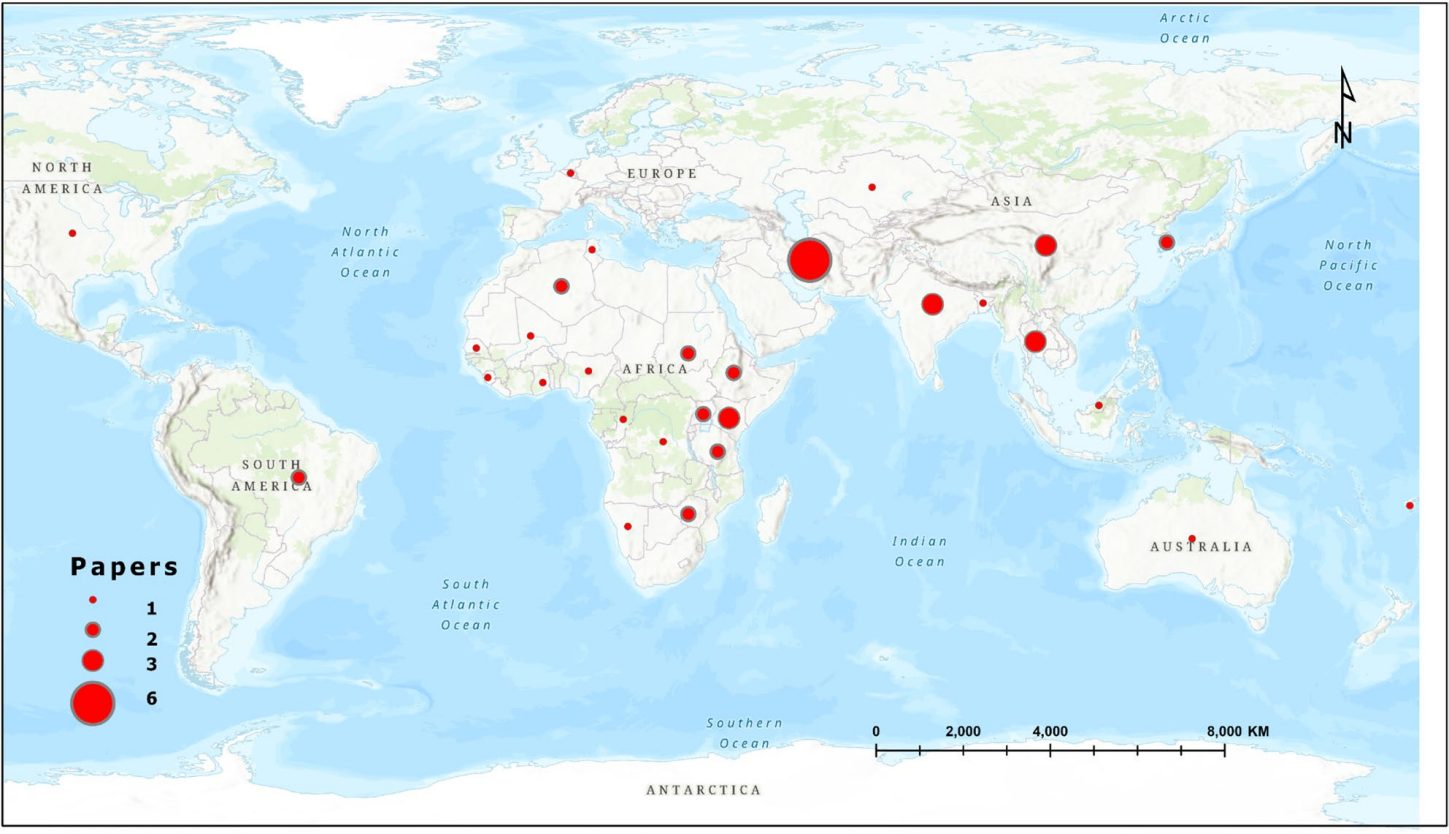
Research paper collection at country level

The geographical distribution of the research effort shows a diverse yet concentrated pattern across countries (Table 1). Iran leads with six publications, followed by China, India, Kenya, and Thailand, each contributing three studies. A cohort of nations including Algeria, Ethiopia, Zimbabwe, Tanzania, Uganda, South Korea, Sudan, and Brazil are each represented by two papers, while all remaining countries contributed a single publication. This global distribution, visualized in Fig. 5, displays the worldwide research interest in zoonotic disease geospatial modelling, while also highlighting the prominence of specific endemic regions and major research hubs in the published literature. The concentration of studies in countries like Iran, Kenya, and Thailand suggests these nations are either focal points for high burden zoonoses or centers of significant methodological development within the field.

**Table 1:** Research papers by country

Country	Frequency	Country	Frequency
Algeria	2	Namibia	1
Ethiopia	2	Senegal	1
Zimbabwe	2	Mali	1
Tanzania	2	China	3
Kenya	3	India	3
Uganda	2	Iran	6
Ghana	1	Kazakhstan	1
Congo	1	Belgium	1
DRC	1	Bangladesh	1
Nigeria	1	Brazil	2
Tunisia	1	Fiji	1
Sierra Leone	1	Sudan	2
Malaysia	1	Thailand	3
Australia	1	South Korea	2
America	1		

#### Distribution of reviewed papers by continent

The continental distribution of the reviewed literature, summarized in Table 2, demonstrates a pronounced focus on regions with high zoonotic disease burdens and active research landscapes. Studies conducted in Africa accounted for 52.2% (*n* = 24) of the reviewed literature, followed by Asia with 21.7% (*n* = 10) and Europe with 13.5% (*n* = 6). Collectively, these regions represented 87.4% (*n* = 40) of the 46 included studies. In contrast, the representation from other continents is markedly lower, with three articles from the Americas, two from Australia, and a single publication from Europe. This geographical concentration demonstrates the research priority placed on understanding zoonotic disease contexts, aligning with the review’s primary scope while reflecting the global distribution of both disease risk and scholarly investment in spatial epidemiological methods.

**Table 2:** Research papers by continent

Continent	Number
Africa	20
Asia	20
Europe	1
America	3
Australia	2

### Summary of the studies reviewed

The final set of 46 studies selected for this systematic review that are summarized comprehensively in Table 3, represents a diverse corpus of research applying geospatial modelling to zoonotic diseases within a One Health framework. This compilation details each study’s retrieval source, full reference, title, year of publication, and the specific geospatial modelling approach employed. As catalogued in the Table 3, the methodological approaches are notably varied, encompassing foundational techniques such as Kernel Density Estimation and spatial autocorrelation analyses, sophisticated machine learning ensembles and Random Forest models, advanced Bayesian spatiotemporal frameworks, and specialized ecological niche modelling algorithms. This extensive methodological spectrum displays the field’s technical evolution and highlights the tailored application of spatial analytics to distinct epidemiological questions and ecological contexts across the included literature (Table 3).

**Table 3:** Final studies included in the systematic review

Retrieved from	Reference	Title	Year	Geospatial Modelling Approach
Science direct	[15]	A Bayesian modeling and retrospective analysis of cutaneous leishmaniasisin the Sahara Desert	2025	Bayesian Modelling
Science direct	[16]	Anthrax in the Amhara regional state of Ethiopia; spatiotemporal analysis and environmental suitability modeling with an ensemble approach	2020	Ensembled Machine learning
Springer	[17]	An exploratory GIS-based method to identify and characterise landscapes with an elevated epidemiological risk of Rhodesian human African trypanosomiasis	2012	Land use land cover classification, Kernel density estimation
Springer	[18]	Analysis of the Spatiotemporal Distribution and Evolutionary Trends of Scrub Typhus in Jiangsu Province from 2006 to 2023	2025	Spatial-temporal hotspot analysis
Springer	[19].	Anthrax hotspot mapping in Kenya support establishing a sustainable two‑phase elimination program targeting less than 6% of the country landmass	2022	Kernel density estimation
Springer	[20]	Drivers and potential distribution of anthrax occurrence and incidence at national and sub‑county levels across Kenya from 2006 to 2020 using INLA	2022	Integrated nested Laplace approximations (INLA)
Springer	[21]	Fascioliasis risk factors and space-time clusters in domestic ruminants in Bangladesh	2017	Kriging
Springer	[22]	Mapping the stability of febrile illness hotspots in Punjab from 2012 to 2019- a spatial clustering and regression analysis	2023	spatial clustering and regression analysis
Springer	[23]	Modelling climate change impacts on the spatial distribution of anthrax in Zimbabwe	2024	General Linear Model (GLM), Multiple Adaptive Regression Spline (MARS), Surface Range Envelope (SRE), Generalized Boosted model (GBM), Random Forest (RF), Classification Tree Analysis (CTA), Flexible Discriminant Analysis (FDA) and Maximum Entropy (MaxEnt)
Springer	[24]	Multi-level analyses of spatial and temporal determinants for dengue infection	2006	Land use land cover Classification, Kulldorff spatial scan statistic
Springer	[25]	Participatory mapping identifies risk areas and environmental predictors of endemic anthrax in rural Africa	2022	Bayesian spatial logit-binomial generalised linear mixed-effects model (GLMM)
Springer	[26]	Prevalence, associated risk factors and satellite imagery analysis in predicting soil-transmitted helminth infection in Nakhon Si Thammarat Province, Thailand	2025	Satellite imagery analysis, CNN, PCA, Heat Maps
Springer	[27]	Spatial analysis and risk mapping of Crimean-Congo hemorrhagic fever (CCHF) in Sub-Saharan Africa	2025	R-INLA (INLA + inlabru packages)
Wiley online library	[28]	Land use gradients drive spatial variation in Lassa fever host communities in the Eastern Province of Sierra Leone	2025	Land use land cover classification
Pubmed	[29]	Mapping as a tool for predicting the risk of anthrax outbreaks in Northern Region of Ghana	2016	Spatial analyst tool
MDPI	[30]	Mapping the Geographic Distribution of Tungiasis in Sub-Saharan Africa	2020	Satellite imagery analysis, resampling, mapping(Arcgis), Dimensionality reduction (PCA), Ecological niche modelling (R)
Google scholar	[31]	Mapping Monkeypox Transmission Risk through Time and Space in the Congo Basin	2012	Satellite imagery analysis(NDVI), Ecological Niche modelling
Science direct	[32]	Integrating remote sensing, GIS, and machine learning for zoonotic cutaneous leishmaniasis modelling	2025	Trend analysis, Remote sensing, Machine learning (RF & XGBoost)
MDPI	[33]	Spatiotemporal Distribution of Tuberculosis in the Oromia Region of Ethiopia: A Hotspot Analysis	2023	Bayesian spatiotemporal modeling, The Getis–Ord Gi statistic
JMIR	[34]	Distribution and Risk Factors of Scrub Typhus in South Korea, From 2013 to 2019: Bayesian Spatiotemporal Analysis	2025	Bayesian spatiotemporal modeling, The Getis–Ord Gi statistic
Google scholar	[35]	Use of geographically weighted logistic regression to quantify spatial variation in the environmental and sociodemographic drivers of leptospirosis in Fiji: a modelling study	2018	Weighted logistic regression
Science direct	[36]	Bayesian spatio-temporal modeling to assess the effect of land-use changes on the incidence of Cutaneous Leishmaniasis in the Brazilian Amazon	2024	Bayesian Spatiotemporal modelling
Science direct	[37]	Bayesian spatio-temporal modelling to assess the role of extreme weather, land use change and socio-economic trends on cryptosporidiosis in Australia, 2001–2018	2021	Bayesian Spatiotemporal modelling
Science direct	[38]	Cutaneous leishmaniasis prevalence and morbidity based on environmental factors in Ilam, Iran: Spatial analysis and land use regression models	2016	Land use regression (LUR) analysis, Inverse Distance weight
Science direct	[39]	Spatiotemporal analysis of brucellosis incidence in Iran from 2011 to 2014 using GIS	2017	The Cochran–Armitage test for linear trends, choropleth maps, hot-spot analysis, and high–low clustering
Google scholar	[40]	A One Health perspective to identify environmental factors that affect Rift Valley fever transmission in Gezira state, Central Sudan	2019	Land use land cover classification, NDVI
Google scholar	[35]	An assessment of risk factors for contracting rabies among dog bite cases recorded in Ward 30, Murewa district, Zimbabwe	2021	Inverse distance weighting (IDW)
Google scholar	[41]	Climate determines transmission hotspots of Polycystic Echinococcosis, a life-threatening zoonotic disease, across Pan-Amazonia	2023	Kriging
Google scholar	[42]	Zoonotic cutaneous leishmaniasis in northeastern Iran: a GIS-based spatio-temporal multi-criteria decision-making approach	2016	Analytical hierarchal process
Google scholar	[43]	Identifying leptospirosis hotspots in Selangor: uncovering climatic connections using remote sensing and developing a predictive model	2024	Spatial autocorrelation analysis (Moran’s I) and Getis-Ord Gi* (hotspot analysis), Support vector machine (SVM), Random Forest (RF), and light gradient boosting machine (LGBM)
Science direct	[44]	Ecological niche modeling as a tool for prediction of the potential geographic distribution of Bacillus anthracis spores in Tanzania	2018	Ecological niche modelling, Maxent algorithm
Science direct	[45]	Forest fire dynamics in India (2005–2022): Unveiling climatic Impacts, spatial Patterns, and interface with anthrax incidence	2024	Kriging
Science direct	[46]	Remote sensing, land cover changes, and vector-borne diseases: Use of high spatial resolution satellite imagery to map the risk of occurrence of cutaneous leishmaniasis in Ghardaïa, Algeria	2014	Land use land cover Classification, risk mapping
Google scholar	[47]	Geographical and temporal distribution of the residual clusters of human leptospirosis in China, 2005–2016	2018	Spatial autocorrelation (Global Moran’s I)
Science direct	[48]	Co-occurrence of leptospirosis and Opisthorchis viverrini infection in cats and their risk factors	2025	Inverse distance weighting (IDW)
Science direct	[49]	Influence of urban expansion on Lyme disease risk: A case study in the U.S. I-95 Northeastern corridor	2022	Land use land cover classification
Science direct	[50]	Identifying high-risk areas for dog-mediated rabies using Bayesian spatial regression	2022	Bayesian spatial regression model
Science direct	[51]	Rabies in Tunisia: A spatio-temporal analysis in the region of CapBon-Nabeul	2021	Kernel Density Estimation (KDE)
Science direct	[52]	Spatial modelling to identify high-risk zones for the transmission of cutaneous leishmaniasis in hyperendemic urban environments: A case study of Mashhad, Iran	2025	GIS MCDM model (TOPSIS)
Google scholar	[53]	Spatial modeling and ecological suitability of monkeypox disease in Southern Nigeria	2022	MaxEnt
Google scholar	[54]	Epidemiological features and spatial-temporal clustering of visceral leishmaniasis in mainland China from 2019 to 2021	2022	Moran’s I and Getis-ORD Gi∗
Google scholar	[55]	Prone Regions of Zoonotic Cutaneous Leishmaniasis in Southwest of Iran: Combination of Hierarchical Decision Model (AHP) and GIS	2019	AHP, Remote sensing
Google scholar	[56]	Spatial analysis to assess the relationship between human and bovine brucellosis in South Korea, 2005–2010	2018	Moran’s I, Bivariate LISA (BiLISA)
Science direct	[57]	Spatial variation and hotspot detection of COVID-19 cases in Kazakhstan, 2020	2020	Average Nearest Neighbour Analysis (ANN), Moran’s I, Getis-Ord Gi* (Hotspot Analysis)
Science direct	[58]	Spatio-temporal analysis of leptospirosis outbreaks during the 2018 kerala floods: Linking flood risk, population density, land use and health infrastructure gaps	2025	Buffer analysis, Getis-Ord Gi*
MDPI	[59]	Developing the Role of Earth Observation in Spatio-Temporal Mosquito Modelling to Identify Malaria Hot-Spots	2022	Remote sensing, Random forest Modelling

### Geospatial modelling techniques used to map zoonotic disease hotspots

In response to the first research question concerning the geospatial modelling techniques employed to map zoonotic disease hotspots, with the frequency of each method (Table 4). Bayesian spatial modelling, Getis–Ord Gi* hotspot analysis, and satellite imagery analysis were the most frequently applied approaches, each used in 6 of the 46 studies (13.0%). Land use/land cover classification and Moran’s I spatial autocorrelation were each applied in 4 studies (8.7%), while all other methods were used in fewer than 7% of studies individually. This was closely followed by land use land cover classification and Moran’s I spatial autocorrelation analysis, each featured in four studies. A wide array of other techniques was also represented, including kernel density estimation, ecological niche modelling, random forest modelling, kriging, inverse distance weighting, maximum entropy algorithms, and analytical hierarchical processes, among others.

**Table 4:** Most used geospatial modelling approaches

Most used geospatial modelling approach	# of times used	Most used geospatial modelling approach	# of times used
Bayesian modelling	6	Maximum entropy algorithm	3
Satellite imagery analysis	6	Heat maps	2
Getis-Ord Gi*	6	Analytical hierarchal process	2
LULC Classification	4	Weighted logistic regression	2
Moran’s hotspot analysis	4	Integrated nested Laplace	2
Kernel density estimation	3	Generalised linear model	2
Kriging	3	Hotspot analysis	2
Inverse distance weight	3	Random forest modelling	3
Ecological niche modelling	3		

### Predictors of zoonotic disease risk: environmental, climatic, and socio-ecological variables

To address the second research question, which examines the predictors used in geospatial models of zoonotic disease, a systematic analysis of environmental, climatic, animal health, and socio ecological variables across the reviewed literature was conducted. The results, summarized comprehensively in Table 5, demonstrate that climatic variables are the dominant predictors, with temperature and precipitation featured in 24 and 22 studies respectively. Population density was the most frequently integrated socio ecological factor, utilized in 17 studies. Additional common predictors included topographic and environmental features such as elevation, soil properties, and proximity to water bodies, each used in multiple studies. Land use data, humidity, and evapotranspiration were also regularly incorporated, while variables reflecting broader socio economic dynamics, such as poverty levels and urbanization trends, were applied less frequently. This distribution of predictors, detailed in Table 5, shows the primary reliance on readily available climatic and remote sensing derived environmental data, coupled with a consistent but secondary integration of key demographic and land use factors to model the complex drivers of zoonotic disease risk. Climatic variables dominated the predictor space, with temperature included in 24 studies (52.2%) and precipitation in 22 studies (47.8%). Socio-ecological variables were less frequently incorporated; population density appeared in 17 studies (37.0%), while poverty indicators and urbanization trends were included in only 2 studies each (4.3%).

**Table 5:** Environmental, climatic, animal health, and socio-ecological variables a commonly used as predictors

Most used predictor	# of times used	Most used predictor	# of times used
Temperature	24	Evapo-transpiration	3
Precipitation	22	Urbanization trend	2
Population density	17	Poverty levels	2
Elevation	9	Wind speed	1
Soil properties	7	Wind direction	1
Land use data	6	Climate change	1
Proximity to water bodies	6	Proximity to farms	1
Humidity	3		

### Integration of one health principles into geospatial modelling frameworks

The systematic evaluation conducted for the third research question demonstrates that current geospatial modelling frameworks integrate One Health principles primarily through the deliberate synthesis of data from human, animal, and environmental domains, achieved by incorporating a diverse suite of predictor variables covering environmental, climatic, animal health, and socio ecological dimensions. Across the 46 studies included in the review, levels of One Health integration varied considerably. Seven studies (15.2%) integrated all three core domains, namely human, animal, and environmental health, within a single geospatial modelling framework. Twenty one studies (45.7%) integrated two domains, most commonly linking environmental drivers with human disease outcomes. Eighteen studies (39.1%) focused on a single domain but demonstrated implicit One Health relevance through spatial contextualization of zoonotic risk. Explicit reference to One Health frameworks was present in 18 studies (39.1%), whereas the remaining studies exhibited implicit alignment based on analytical integration rather than terminology.

### Factors that hinder effective geospatial modelling of zoonotic diseases

Several recurrent factors were identified as constraining the effectiveness of geospatial modelling of zoonotic diseases (Table 6). Data availability and data quality constraints were the most frequently reported limitations, collectively cited by 19 studies (41.3%). Limitations related to temporal coverage, including short data collection periods and restricted time spans of available datasets, were reported in 5 studies (10.9%). Constraints associated with spatial resolution, particularly the lack of fine scale spatial data, were noted in 4 studies (8.7%). Methodological challenges, including issues related to model validation, high correlation among predictor variables, small sample sizes, and unanalysed clustering effects, were identified in 6 studies (13.0%). Additional limitations included data reporting biases, reliance on secondary data susceptible to bias, and underestimation of actual incidence rates, which were collectively reported in 5 studies (10.9%). Overall, 33 of the 46 studies (71.7%) explicitly acknowledged at least one limitation affecting the robustness of geospatial modelling outcomes.

**Table 6:** Factors that hinder effective Geospatial modelling of zoonotic diseases

Challenge	Frequency	Challenge	Frequency
Data availability	15	Data quality	4
Time taken in data collection	3	Use of few environmental variables	2
Data reporting challenges	2	Model related challenges	2
Secondary data susceptible to bias	1	Clustering factors not analyzed	1
Restricted period of the available data	1	Small sample size	1
Lack of finer spatial resolution data	1	High correlation among predictor variables	1
Underestimating the actual incidence rates	1	Data validation	1

### Most mapped zoonotic diseases

The analysis of disease focus across the reviewed literature reveals a distinct concentration on a subset of zoonoses, with *Cutaneous Leishmaniasis* being the most frequently mapped disease, featured in nine studies. Anthrax was the second most prevalent, examined in eight studies, followed by *leptospirosis* in five studies. Other diseases that were the subject of multiple investigations include rabies, scrub typhus, and brucellosis. A wider array of zoonotic conditions, including tuberculosis, COVID 19, and various vector borne and parasitic diseases such as Rift Valley fever, dengue, and Lyme disease, were each represented by a single study (Table 7). This distribution indicates a strong research emphasis on diseases with significant cutaneous, environmental, or livestock associated transmission pathways, reflecting both their public health burden and their suitability for modelling with available environmental and climatic predictor variables.

**Table 7:** Most mapped zoonotic diseased

Disease	Frequency	Disease	Frequency
*Cutaneous Leishmaniasis*	9	Helminth	1
Anthrax	8	Fascoliasis	1
Leptospirosis	5	Trypanosomiasis	1
Rabies	3	Cryptosporidiosis	1
Scrub typhus	2	*Polycystic Echinococcosis*	1
Brucellosis	2	Dengue	1
Tuberculosis	1	Lyme disease	1
Covid 19	1	Tungiasis	1
Malaria	1	Lassa fever	1
Hemorrhagic fever	1	Febrile illnesses	1
Rift valley fever	1		

## Discussion

This systematic review provides a comprehensive synthesis of how geospatial modelling has been applied to identify zoonotic disease hotspots within a One Health framework. Drawing on evidence from 46 peer reviewed studies, the review elucidates dominant methodological approaches, prevailing data integration practices, and recurring constraints that collectively shape the current state of the field (Sect. Distribution of papers to Factors that hinder effective geospatial modelling of zoonotic diseases). Taken together, the findings underscore the growing centrality of spatial analytics in disentangling the complexity of zoonotic disease systems, while also revealing persistent structural and methodological gaps that constrain the effective translation of geospatial insight into integrated surveillance and targeted intervention strategies.

A clear temporal expansion of the literature was observed, with nearly half of all included studies published during the most recent period from 2023 to 2025 (Sect. Distribution of reviewed papers based on year; Fig. 2). This surge reflects an intensifying recognition of the need for spatially explicit and interdisciplinary approaches to zoonotic disease management, particularly in the wake of the COVID-19 pandemic. The increased adoption of geospatial modelling aligns closely with global health priorities that emphasize early warning, preparedness, and prevention of emerging infectious diseases through operationalization of the One Health paradigm [2]. Despite this growth, the geographic distribution of studies remains uneven. Africa, Asia, and Europe together accounted for 87.4% of the reviewed literature (Sect. Summary of the studies reviewed; Fig. 3), with research activity concentrated in a limited number of countries such as Iran, Kenya, and Thailand. This clustering suggests that geospatial modelling efforts are often driven by a combination of high local disease burden and the availability of technical capacity in spatial epidemiology. At the same time, the relative paucity of studies from several high risk African settings highlight enduring disparities in research infrastructure, access to spatial data, and investment in analytical capacity.

While Bayesian spatial models, machine learning approaches, and satellite-based analyses offer powerful tools for advancing zoonotic disease mapping, each is associated with important limitations that warrant careful consideration. Bayesian models, although well suited to data-sparse environments due to their ability to incorporate prior information and quantify uncertainty, can be sensitive to prior specification and may be computationally intensive, potentially limiting their accessibility and reproducibility across settings [3]. Machine learning methods excel at capturing nonlinear relationships and complex interactions among predictors, yet their often limited interpretability can hinder mechanistic understanding and reduce transparency for public health decision making, particularly when model outputs are used to inform policy. In addition, many machine learning models exhibit strong dependence on large, high quality training datasets, raising concerns about robustness and performance in data constrained contexts [7]. On the other hand, the satellite-based approaches provide valuable environmental information at broad spatial scales and help overcome gaps in ground-based surveillance. However, their effectiveness is constrained by the spatial and temporal resolution of available imagery and by challenges in translating remotely sensed proxies into epidemiologically meaningful indicators. Furthermore, the transferability of models relying heavily on remotely sensed data across ecological and socio-political contexts remains uncertain, particularly where land use patterns, reporting systems, and human–animal interactions differ substantially. Recognizing these limitations is essential for ensuring that methodological innovation is accompanied by critical evaluation of suitability, interpretability, and generalizability across diverse One Health contexts [1].

Analysis of predictor variables further highlights important imbalances in current modelling practice. Climatic variables, particularly temperature and precipitation, dominated the predictor space, appearing in more than half of the reviewed studies (Sect. 3.4; Table 4). This emphasis is consistent with extensive evidence linking climate to vector ecology, pathogen survival, and host distribution. However, socio ecological variables remain comparatively underrepresented. While population density was frequently included as a proxy for human exposure, more nuanced structural determinants such as poverty, livestock mobility patterns, and access to health services were incorporated in only a small fraction of studies. This skew toward biophysical drivers may result in models that effectively identify ecological suitability for pathogen transmission but inadequately represent the social and behavioural processes that ultimately shape spillover risk and outbreak amplification. Consequently, model outputs may have limited utility for informing equitable and context specific intervention strategies [6].

A central contribution of this review lies in its assessment of how One Health principles have been operationalized within geospatial modelling frameworks. The degree of integration across domains varied markedly across studies. Full integration of human, animal, and environmental domains was observed in only 15.2% of studies (Sect. 3.5; Table 5), indicating that comprehensive One Health operationalization remains relatively rare. More commonly, studies implemented partial integration, typically linking environmental predictors with human disease outcomes. Where multi domain integration was achieved, it enabled identification of spatial convergence zones in which environmental suitability, animal reservoir presence, and human vulnerability intersect, reflecting core One Health systems thinking [1]. The use of spatiotemporal modelling approaches further strengthened this integration by capturing dynamic interactions at the human animal environment interface, supporting assessment of seasonal variability, ecological change, and outbreak risk following environmental disturbances [8]. These advances demonstrate the analytical potential of geospatial tools to support coordinated, multisectoral decision making when integration is meaningfully achieved.

Despite these methodological advances, the review identifies substantial constraints that continue to limit the effectiveness and scalability of geospatial modelling for zoonotic diseases. Data availability and data quality challenges were the most frequently reported limitations, affecting more than 40% of studies (Sect. 3.6; Table 6). Fragmentation of data systems across human health, animal health, and environmental monitoring remains a critical barrier to integrated analysis, undermining the core objectives of the One Health approach. These challenges were particularly pronounced in sub-Saharan Africa and other low- and middle-income settings, which constitute a substantial proportion of the reviewed literature, although similar limitations were also reported in studies from other regions [1]. Addressing these constraints will require sustained investment in integrated digital surveillance infrastructure, harmonized data standards, and cross sectoral data sharing mechanisms that facilitate timely and high-resolution spatial analysis.

## Conclusion and recommendations

This systematic review demonstrates that geospatial modelling has become an essential tool for identifying zoonotic disease hotspots within a One Health framework, offering valuable insights into spatial risk patterns and supporting integrated surveillance. Evidence from 46 peer reviewed studies shows that geospatial approaches are most effective when environmental and epidemiological data are combined, yet their broader utility remains constrained by persistent data and integration challenges. Limitations in data availability, quality, spatial resolution, and temporal coverage continue to restrict model robustness, underscoring the need for interoperable surveillance platforms that link human, animal, and environmental information across sectors. At the same time, the underrepresentation of socio ecological drivers in current models highlights the importance of expanding predictor sets to better capture social vulnerability, livestock dynamics, and health system access. Although One Health principles are increasingly referenced, comprehensive integration across human, animal, and environmental domains remains uncommon, indicating that methodological innovation must be matched by institutional coordination and cross disciplinary collaboration. Addressing these interconnected challenges will be critical for enabling geospatial modelling to support proactive, equitable, and evidence informed zoonotic disease prevention and control. 

## Supplementary Information

Below is the link to the electronic supplementary material.

Supplementary Material 1

Supplementary Material 2

## Data Availability

No datasets were generated or analysed during the current study.
