# Supplementary material for: Geospatial modelling for zoonotic disease hotspot identification within a One Health framework: a systematic review
Source: One Health Outlook. 2026 Jan 27;8:7. doi: 10.1186/s42522-026-00194-8 (PMC12857150; doi:10.1186/s42522-026-00194-8)
Supplement: Supplementary file 1 — Supplementary Material 1 [file 42522_2026_194_MOESM1_ESM.docx]

Supplementary Table S1. Database-specific search strategies

| Database | Search string | Filters / limits |
| --- | --- | --- |
| ScienceDirect | (“zoonotic disease” OR zoonosis OR zoonoses) AND (“geospatial modeling” OR GIS OR “spatial analysis” OR “hotspot mapping” OR “spatial epidemiology” OR “remote sensing”) AND (“One Health”) | Article type: Research articles; Language: English; Years: 2000–2025 |
| PubMed | (“zoonoses”[MeSH] OR “zoonotic diseases”) AND (“spatial epidemiology” OR GIS OR “hotspot analysis”) AND (“One Health”) | Humans/Animals; English; 2000–2025 |
| SpringerLink | (“One Health” AND “spatial analysis”) AND (zoonotic OR zoonoses) | Article type: Article; English |
| MDPI | (“zoonotic disease” AND “geospatial modelling” AND “One Health”) | Journals only; English |
| Wiley Online Library | (“zoonotic disease” AND GIS AND “One Health”) | Research articles; English |
| JMIR | (“zoonotic” AND “spatial” AND “One Health”) | All article types |
| Google Scholar | “zoonotic disease” AND (“GIS” OR “spatial analysis”) AND “One Health” | First 200 results sorted by relevance |
| ResearchGate | Keyword-based search using predefined terms | Peer-reviewed articles only |

**Supplementary Table S2. Quality appraisal tool and scoring criteria**

| Domain | Criterion | Score 0 | Score 1 | Score 2 |
| --- | --- | --- | --- | --- |
| Study design | Clear objectives and rationale | Absent/unclear | Partially stated | Clearly stated |
| Data quality | Description of spatial and epidemiological data | Poorly described | Adequately described | Comprehensive and transparent |
| Modelling rigor | Appropriateness of geospatial methods | Inappropriate/unclear | Appropriate but limited | Appropriate and rigorous |
| One Health integration | Cross-domain data integration and interpretation | Single-domain | Partial integration | Explicit or analytical integration |
