# Supplementary material for: Geospatial modelling for zoonotic disease hotspot identification within a One Health framework: a systematic review
Source: One Health Outlook. 2026 Jan 27;8:7. doi: 10.1186/s42522-026-00194-8 (PMC12857150; doi:10.1186/s42522-026-00194-8)
Supplement: Supplementary file 2 — Supplementary Material 2 [file 42522_2026_194_MOESM2_ESM.docx]

**Supplementary Material**

Supplementary Table S1. Database-specific search strategies

| Database | Search string | Filters / limits |
| --- | --- | --- |
| ScienceDirect | (“zoonotic disease” OR zoonosis OR zoonoses) AND (“geospatial modeling” OR GIS OR “spatial analysis” OR “hotspot mapping” OR “spatial epidemiology” OR “remote sensing”) AND (“One Health”) | Article type: Research articles; Language: English; Years: 2000–2025 |
| PubMed | (“zoonoses”[MeSH] OR “zoonotic diseases”) AND (“spatial epidemiology” OR GIS OR “hotspot analysis”) AND (“One Health”) | Humans/Animals; English; 2000–2025 |
| SpringerLink | (“One Health” AND “spatial analysis”) AND (zoonotic OR zoonoses) | Article type: Article; English |
| MDPI | (“zoonotic disease” AND “geospatial modelling” AND “One Health”) | Journals only; English |
| Wiley Online Library | (“zoonotic disease” AND GIS AND “One Health”) | Research articles; English |
| JMIR | (“zoonotic” AND “spatial” AND “One Health”) | All article types |
| Google Scholar | “zoonotic disease” AND (“GIS” OR “spatial analysis”) AND “One Health” | First 200 results sorted by relevance |
| ResearchGate | Keyword-based search using predefined terms | Peer-reviewed articles only |

**Supplementary Methods: Operational Criteria for Assessing One Health Integration**

One Health integration was operationalized using a structured assessment framework applied

during both screening and quality appraisal. Each study was evaluated against three domains: (i)

human health (e.g. human case data, population vulnerability), (ii) animal health (e.g. livestock

or wildlife data, reservoir or vector information), and (iii) environmental or climatic factors (e.g.

land use, climate variables, ecological suitability).

Studies were categorized as explicitly aligned with One Health if they met at least two of these

domains and explicitly framed the analysis within a One Health or cross-sectoral context.

Implicit alignment was assigned to studies that integrated at least two domains analytically

within geospatial models but did not explicitly reference One Health terminology.

For each study, integration was assessed independently by two reviewers. Discrepancies in

classification were resolved through discussion and consensus. This structured approach was

designed to minimize subjectivity and ensure consistent application of One Health principles

across the reviewed literature.

**Supplementary Table S2. Quality appraisal tool and scoring criteria**

| Domain | Criterion | Score 0 | Score 1 | Score 2 |
| --- | --- | --- | --- | --- |
| Study design | Clear objectives and rationale | Absent/unclear | Partially stated | Clearly stated |
| Data quality | Description of spatial and epidemiological data | Poorly described | Adequately described | Comprehensive and transparent |
| Modelling rigor | Appropriateness of geospatial methods | Inappropriate/unclear | Appropriate but limited | Appropriate and rigorous |
| One Health integration | Cross-domain data integration and interpretation | Single-domain | Partial integration | Explicit or analytical integration |
